# Needs, preferences, and patient participation for a randomized controlled trial on postneoadjuvant complete tumor response: A qualitative study of patients with esophageal cancer

**DOI:** 10.1007/s00520-024-08845-0

**Published:** 2024-09-11

**Authors:** Manuel Czornik, Joachim Weis, Andrea Kiemen, Claudia Schmoor, Julian Hipp, Jens Hoeppner

**Affiliations:** 1 Department of Psychiatry and Psychotherapy/Division for Interventional Biological Psychiatry, Faculty of Medicine and Medical Center, University of Freiburg, Freiburg, Germany; 2 Endowed Professorship Self-Help Research, Comprehensive Cancer Center, Faculty of Medicine and Medical Center, University of Freiburg, Freiburg, Germany; 3 Department of Clinical Research, University of Basel, c/o University Hospital Basel, Basel, Switzerland; 4 Clinical Trials Unit, Faculty of Medicine and Medical Center, University of Freiburg, Freiburg, Germany; 5 Department of General Surgery, Faculty of Medicine and Medical Center, University of Freiburg, Freiburg, Germany; 6 Department of Surgery, University Medical Center OWL – Campus Lippe, Detmold, Germany

**Keywords:** Esophageal cancer, Patient participation, Psycho-social needs, Active surveillance, Esophagectomy

## Abstract

**Purpose:**

For patients with clinical complete response of non-metastatic esophageal cancer (EC) after neoadjuvant chemoradiotherapy (nCRT) or neoadjuvant chemotherapy (nCT), the two treatment options obligate postneoadjuvant surgery as the current standard treatment (surgery on principle) versus active surveillance with surgery as needed only in recurring loco-regional tumor as a possible future alternative or standard exist. Since these treatments are presumably equivalent in terms of overall survival, patient-centered information can encourage the discussion with the treating physician and can make it easier for patients to make trade-offs between the advantages and disadvantages of the treatment alternatives in a highly distressed situation.

**Methods:**

A qualitative prospective cross-sectional study was conducted to create patient-centered information material that is based on patients’ preferences, needs, and concerns regarding the two treatment options, and to investigate the potential participation in a consecutive randomized controlled trial (RCT). Therefore, EC patients (*N* = 11) were asked about their attitudes.

**Results:**

Concerns about the surgery and possible postoperative impairments in quality of life were identified as most mentioned negative aspects of surgery on principle, and recurrence and progression fear and the concern that surgery cannot be avoided anyways as most named negative aspects of surgery as needed. In regard to the participation in an RCT, making a contribution to science and the hope that the novel therapy would be superior to the established one were relevant arguments to participate. On the other hand, the lack of a proactive selection of treatment was named an important barrier to participation in an RCT.

**Conclusion:**

The importance of adapting medical conversations to the patients’ lack of expertise and their exceptional cognitive and emotional situation is stressed. Results of this study can be used to improve patient-centered information and the recruitment of patients in RCTs in cancer.

## Introduction

Esophageal cancer (EC), one of the most lethal and least studied cancers worldwide, has lately gained more attention due to significant improvements in survival rates resulting from recent advances in diagnosis, staging, and treatment [1]. The diagnosis includes two types of cancers arising from the esophagus or the gastroesophageal junction—esophageal squamous cell carcinoma (ESCC) and esophageal adenocarcinoma (EAC) [2]. Worldwide, more than 450,000 people are affected by EC, with rapidly growing incidence [3]. The lifetime risk is expected to be 0.8% for men and 0.3% for women. The mean age at diagnosis is 67 years, and the risk of falling ill increases with age [2, 4]. Due to these numbers, the aggressive nature, and a poor survival rate, EC is the sixth leading cause of death from cancer worldwide [5, 6].

To date, the western standard treatment for most patients with non-metastatic EC after neoadjuvant chemotherapy (nCT) or chemoradiotherapy (nCRT) is a surgical resection of the affected part of the esophagus [7–9]. For patients with clinical complete response to neoadjuvant chemoradiotherapy (cCR), active surveillance with surgery only if needed may be an equivalent option regarding survival [9–12]. The benefit of this procedure is the avoidance of potentially harmful major surgery, thereby reducing the complication rate and length of hospital stay and increasing quality of life (QoL). This results in reduced treatment costs and faster return to baseline health-related QoL and socioeconomic productive work life for patients. However, on the other side, in active surveillance with surgery as needed, patients have to adhere to regular follow-up testing and live with the uncertainty of not having removed the tissue affected by cancer [13].

In this situation of presumably equivalent treatment alternatives regarding survival, patient-centered information can encourage discussion with the treating physician and make it easier for patients to make trade-offs between the advantages and disadvantages of the treatment options in a highly distressed situation [14]. This understanding is the prerequisite for informed and shared decision-making (SDM) in general healthcare and can improve the recruitment process and consent to randomization in radomized controlled trials (RCTs) [15]. Although SDM is not the designated process in RCTs, value clarification is important because the decision to take part in one is also driven by subjective and intuitive behavior (e.g., feelings of discomfort, vulnerability, and other intuitive and affect-laden responses) [15–17]. Patient-centered trial information materials, also refered to as decision aids, should include evidence-based information on disease and treatment options, postoperative mortality and morbidity, intermediate and long-term outcomes, side effects, and burdens to daily life of respective treatment options [18].

The aim of this qualitative interview study was the investigation of EC patients’ information needs, preferences, and values in terms of the two treatment options “surgery as needed” and “surgery on principle.” Thus, the gained knowledge shall contribute to a better understanding of patients’ decision-making and attitudes towards participation in a RCT. To address these aims, both EC patients and medical experts were interviewed. In this paper, we focus on the results of the patients’ interviews only. Capturing the opinions of affected patients becomes particularly important when considering evidence that physicians and patients differ substantially in their attitudes towards different treatment options [19, 20].

**Fig. 1:**
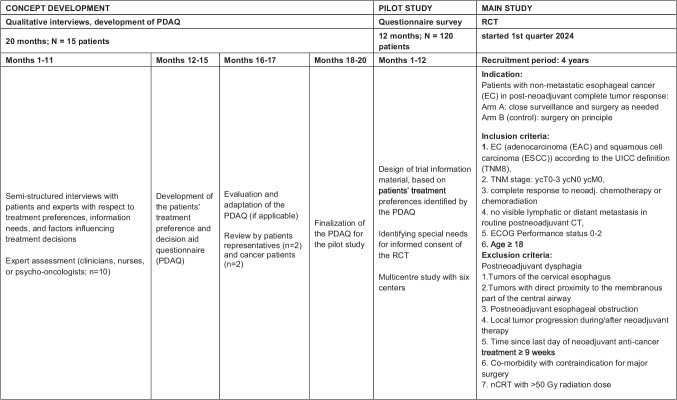
Overview of the different ESORES phases. Note: CT, computer tomography; EAC, adenocarcinoma; EC, esophageal cancer; ECOG, Eastern Cooperative Oncology Group; ESCC, squamous cell carcinoma; nCRT, neoadjuvant chemoradiotherapy; PDAQ, preference and decision aid questionnaire; RCT, randomized controlled trial

This qualitative study was conducted within the concept development phase as the first part of a main study (ESORES RCT) which is aimed to investigate differences in outcomes in EC patients treated with surveillance and surgery as needed only in persisting or recurring locoregional tumor versus surgery on principle. The primary hypothesis is that the overall survival in a protocol with clinical identification and surveillance of complete response (CR) with surgery only as needed is non-inferior compared to surgery on principle. It is hypothesized that surveillance of CR with surgery as needed is superior with regard to morbidity and quality of life. The study was funded by the German Federal Ministry of Education and Research within the research program Nationale Decade against Cancer. Based on the results of the interviews, a Preference and Decision Aid Questionnaire will be designed in the first part of the project for the quantitative evaluation of patients’ preferences in the consecutive pilot study. The gained results will be used for the creation of patient-centered information material for use in the subsequent RCT [21]. An overview of the different phases of the ESORES trial is shown in Fig. 1. This manuscript focuses on the results of the qualitative patient interviews.

## Materials and methods

### Interview guide

The semi-structured interview guide was developed step by step following an extensive literature review, consultations with two representatives of cancer support groups, and pretest interviews with two cancer patients. Interviews with representatives and cancer patients were conducted using the think-aloud protocol, a method in which participants are asked to verbalize their thoughts and express whatever comes to their mind, including their actions and feelings, as they go through the preliminary interview guide [22]. The aim was to gain insight into the participants’ cognitive processes.

Initially, a preliminary interview guide was developed after an extensive literature search and was discussed within the team. Subsequently, an updated version of the guide was created and presented to a representative or cancer patient. After incorporating their feedback, the revised version was then discussed with the next representative or cancer patient. This iterative process continued until feedback from all four external sources was incorporated, and the entire team approved the final interview guide.

### Methods

In a qualitative cross-sectional study, interviews with patients were conducted by psychologists trained in qualitative interviewing techniques using the interview guide (~ 30 min). Eligibility criteria for the patients in this part of the study were the diagnoses of EAC or ESCC according to the definition of the Union for International Cancer Control (UICC) (TNM8, ycT0-3 ycN0 ycM0). Inclusion required the completion of nCT or nCRT regardless of the outcome of the neoadjuvant therapy. Interviews were conducted either pre- or post-surgery. Exclusion criteria were the unavailability of written consent, gastric cancer, tumors of the cervical esophagus, or comorbidity with contraindication for major surgery.

Patients were asked about their information needs, treatment preferences, and the processes of decision-making. The interview also focused on worries, concerns, and their attitudes towards participation in an RCT. Additionally, basic demographic data regarding gender, age, ethnicity, highest level of education, and family status were assessed. Medical data, including time since diagnosis, current health status, clinical stage (cT/cN category), tumor type, pathological stage (pT/pN category and tumor regression staging), and previous and current medical treatment, were taken from medical records.

Recruitment of the interviewees was performed between October 2020 and February 2021 at the Department of General Surgery and the Interdisciplinary Tumor Center of the Medical University Freiburg. Patients with EC were selected by the physicians according to the qualitative concept of purposive sampling, which allows a certain variety of individuals within the frame of the inclusion criteria with respect, e.g., to sex, age, or educational level. Patients’ screening for eligibility was performed by the responsible physician at the Medical Center University of Freiburg. The number of patients to be interviewed was based on the qualitative criterium of saturation on the one hand and the availability of patients within a certain time frame on the other. For this purpose, the interviews are analyzed consecutively until a certain thematic saturation is reached with respect to the research questions. We reached this criterium with a number of 11 patients.

The study protocol was approved by the Ethical Committee of the Medical University Freiburg (No. 20–1037) and was conducted in accordance with the Declaration of Helsinki (DRKS00022050).

### Analysis

The recorded interviews were transcribed verbatim and anonymized. All qualitative analyses were performed at the Professorship Self-help Research of the Interdisciplinary Tumor Center, Medical University Freiburg. Two psychologists independently coded and analyzed the transcripts using MAXQDA [23], implying content-structuring qualitative content analysis according to Kuckartz [24] to identify themes and subthemes. Compared with other approaches of content analysis, the method of Kuckarts enables to analyze not only manifest but also latent contents. In addition, the categories can be created deductively, inductively, or deductively-inductively. First, main categories were assigned according to the interview guide following a deductive approach. Secondly, sub-categories were inductively developed and differentiated from the interview material. Thirdly, the two coders compared and discussed their resulting category systems until they finally agreed on the final category system. Then, the interview material was revised with the final category system. Lastly, anchor quotes were assigned to the codes. Statistical analyses were confined to descriptive analyses.

## Results

A total of 11 EC patients underwent qualitative interviews at different stages of their cancer treatment. The patient sample consisted of nine males (82%) and two females (18%). The patients’ age ranged from 33 to 82 years, with a mean age of 60.2 years (*SD* = 16.2). Six patients were interviewed preoperatively (55%) an average of 34.2 days before surgery (*SD* = 23.7), and five patients (45%) were interviewed postoperatively an average of 91.2 days after surgery (*SD* = 126.8). The diagnosis of eight patients was EAC (72.7%), and three patients had received the diagnosis ESCC (27.3%). Regarding neoadjuvant therapy, seven patients (63.6%) received nCT, and four patients (36.4%) nCRT. Details of the patients can be found in Tables 1 and 2.

**Table 1:** Patients’ demographic data

Patient	Diagnosis	Age	Gender	Highest level of education	Marital status
P01	EAC	79	Male	High school	Married
P02	EAC	59	Male	University degree	Divorced
P03	EAC	33	Male	High school	Unmarried
P04	EAC	57	Male	Secondary school	Unmarried
P05	ESCC	77	Male	Secondary school	Divorced
P06	EAC	59	Female	Secondary school	Unmarried
P07	EAC	82	Male	Primary school	Married
P08	EAC	61	Male	University degree	Married
P09	ESCC	52	Male	Secondary school	Unmarried
P10	EAC	35	Male	University degree	Unmarried
P11	ESCC	68	Female	University degree	Unmarried

**Table 2:** Patients’ medical data

Patient	Neoadjuvant therapy	Time to/since surgery	Surgical method	Pre-therapeutical stage	Post-therapeutical stage	pCR
P01	nCT (FLOT)	125 days **post**	Open esophagectomy	uT3uN0cM1	ypT3ypN3ycM0	No
P02	nCRT (CROSS)	71 days **pre**	HMIE	uN1cM0	ypT0ypN0ycM0	Yes
P03	nCT (FLOT)	43 days **pre**	HMIE + liver resection	uT4uN1cM1	ypT3ypN2ycM1	No
P04	nCT (FLOT)	16 days **pre**	Open esophagectomy	uT4uN1cM0	ypT3ypN3ycM0	No
P05	nCRT (CROSS)	28 days **pre**	HMIE	uN1cM0	ypT0ypN0ycM0	Yes
P06	nCT (FLOT)	13 days **pre**	HMIE	uT3uN0cM0	ypT0ypN0ycM0	Yes
P07	nCT (FLOT)	**Pre** (not scheduled)	Planned HMIE	uT3uN1cM0	-	-
P08	nCT (FLOT)	15 days **post**	HMIE	uT3uN0cM0	ypT2ypN0ycM0	No
P09	nCRT (CROSS)	300 days **post**	HMIE	uT3uN1	ypT0ypN0ycM0	Yes
P10	nCT (FLOT)	8 days **post**	HMIE	uT3uN1cM0	ypT3ypN0ycM0	No
P11	nCRT (CROSS)	8 days **post**	HMIE	uT3uN1cM0	ypT0ypN0ycM0	Yes

*EAC* esophageal adenocarcinoma, *ESCC* esophageal squamous cell carcinoma.

*FLOT* fluorouracil, leucovorin, oxaliplatin, and docetaxel; *HMIE* hybrid minimally invasive esophagectomy; *nCRT* neoadjuvant chemoradiotherapy; *nCT* neoadjuvant chemotherapy; *pCR* pathologic complete response.

The analysis of patient interviews led to the formation of 306 initial codes with seven consistent themes of negative and positive aspects of surgery on principle, negative and positive aspects of surgery as needed, information needs, decision-making, and participation in an RCT. An overview of the main themes and categories is shown in Table 3.

**Table 3:** Main themes and categories

Theme	Category
Negative aspects of surgery on principle	
Positive aspects of surgery on principle	Concerns about the surgery
	Postoperative impairments in quality of life
Negative aspects of surgery as needed	Feeling of security due to the removal of the tumor
	Postoperative pain not that important
Positive aspects of surgery as needed	Recurrence fear and progression fear
	Concern that surgery cannot be avoided
	Avoid surgery
	Both options remain possible
Information needs	
	Technical terms
	Limited receptivity
Decision-making	
	Advice
	Trust in the doctor/the clinical center
Participation in an RCT	Arguments for RCT participation
	Arguments against RCT participation

### Negative aspects of surgery on principle

One of the most mentioned reasons against the surgery on principle by the patients was the *fear of surgery*. Specifically, patients expressed fear regarding unpredicted or rare complications of the operation, not waking up from anesthesia, and the physiological strains associated with surgery. Some patients’ concerns were also linked to the intensive care unit environment and the potential situations they might encounter or witness there.

*Well, first of all, the typical fears about surgery of not waking up or facing complications, for which you have signed that you acknowledge them but hope will not occur. Those were the biggest fears and worries about it.* (Patient #8)

Another frequently mentioned topic with regarding negative aspects of surgery on principle was *postoperative impairments in QoL*. This included pain and decreased physical capability following the operation. However, the majority of concerns were releated to diet. Many patients associated these concerns with physical changes resulting from the surgery, particularly regarding food intake. These mentioned symptoms included heartburn, nausea, fear of receiving food through a tube, and fatigue following meals.

*After each meal, I could take a nap, simply because I am so exhausted, even now. After each meal, I could sleep because I am so tired.* (Patient #9)

In addition to these physiological changes, many patients also feared the social consequences associated with dietary alterations. They expressed discomfort, feelings of shame, and fear of social rejection and isolation when eating in restaurants, stemming from the decreased size of the stomach.

*If I go to a steak house, they will laugh at me when I ask for a children’s or senior’s portion, or something like that. Or leave out the side dishes; I only want the piece of meet. The rest doesn’t fit in.* (Patient #9)

### Positive aspects of surgery on principle

Despite the concerns and fears about surgery and its consequences, many EC patients also placed hope in this treatment approach. They desired immediate and complete removal of the tumor and anticipated a newfound and more courageous sense of life that accompanies it. For them, the operation is linked to reducing the burdensome feeling established by the diagnosis.

*I'll put it this way, when you know that there's something in you that doesn't actually belong there, you always have a bit of an oppressive feeling in the back of your mind. It is, how should I say, you have normal - you have a normal quality of life, but you always know that there is still something in your body that actually - in principle, does not belong there; And a certain queasy feeling always remains.* (Patient #2)

Regarding the physical pain experienced directly after esophagectomy and in the following days and weeks, a majority emphasized that it is not particularly relevant. They stressed that even if the *pain* is intense during that period, if it stops hurting in the long run, this aspect can be overlooked. This was especially true for patients who already endured significant pain during the course of their disease.

*The pain I had before, that I had in my throat, was so terrible that it probably couldn't be worse.* (Patient #5)

### Negative aspects of surgery as needed

Many patients associated the surgery-only-as-needed approach with an increased risk of further cancer spread. They described the *fear of progression* as a feeling akin to a “ticking time bomb” inside their bodies, causing considerable insecurity.

*The biggest concern would be that the cancer would still be in the body. That means, wherever it may stray, you discover weeks or months later that things have aggravated. In the worst case.* (Patient #8)

Some patients expressed the view that since esophagectomy may still be necessarywith the surveillance approach, it might be better to undergo it immediately. Thus, patients do not have to delay surgery, sparing themselves surveillance appointments and the feeling of tumor tissue remaining in their bodies.

*You could check it regularly and you're done, but that's the joke. If it gets worse, then you have to operate. That’s the problem. Then why don't you say right away, we'll get the rubbish out and done, and the issue is over, right?* (Patient #5)

### Positive aspects of surgery as needed

Nearly all patients emphasized that the surveillance approach includes the possibility of avoiding surgery and its potential negative consequences. These negative consequences include risks during and after the operation, as well as long-term implications and impairments.

*So, I'll say it like this, if it [the tumor] would behave calmly, then I would prefer that [surveillance with surgery as needed], because every intervention is also a strain on the body. I would say, okay, let's see what happens. So that would be my method, I have to admit.* (Patient #2)

One patient mentioned that unlike surgery on principle, surveillance with surgery only as needed allows one not to commit immediately to one treatment and decide later if esophagectomy is desired. Patient can take their time and wait until the clinical picture, or the own preferences change to be more certain about their decision.

*Yes, I would recommend that to those [who are in this situation]. You should consider that. And if it gets worse, you can still operate, can’t you?* (Patient #5)

### Information needs

For some patients, addressing comprehension problems during the information process was crucial. These issues included discussions with medical experts, but it seemed to be a bigger problem for patients regarding written letters or documents such as medical reports. Nearly all attributed this to the large number of technical terms.

*Well, I mean, this information with all these foreign words, that's a problem. When you get a doctor's letter like that, you don't understand it at all. I ask myself quite often, couldn't they also write it in a way that you understand? No? Words are thrown at you, you can hardly read, not to mention that you don't understand them at all, right? There should be more effort.* (Patient #7)

In addition to content-related problems, some patients also attributed comprehension issues to the situation in which the information process takes place. They cited factors such as cognitive impairments due to the disease, medication, or chemotherapy, as well as being shocked by the diagnosis or anxious about possible answers that might follow uncomfortable questions. Moreover, patients reported that time pressure and sometimes also the time of the day made it difficult for them to follow discussions and ask important questions.

*There are many questions that come to mind. I really have trouble remembering questions. Because I don't know, is it the medication or something else, or am I already suffering from Alzheimer's or something. To concentrate... and then pick up the thread of the conversation again.* […] *Everything has been said, but it's like the round this morning. So quickly. I just woke up. I didn't even see what they wanted or anything. Later, I told the nurse I have so many questions on my mind, I was totally exhausted this morning, and by then, they had all disappeared again. I have so many questions. I have a follow-up appointment next week and now I've made a list. […] A little more time for the patients… that's it. But other patients said to me, surgeons don't have time.* (Patient #9)

### Decision-making

A large proportion of patients reported discussing the diagnosis and possible treatment options with their family and friends. Typically, this involved their closest family and friends, but if the person had medical knowledge, also less afilliated persons were consulted.

*With friends, with family. So in my case, I have friends who are doctors that I could discuss this with.* (Patient #1)

For a few patients, it was also important to discuss the diagnosis and availabte therapies with their general practitioner, a second independent doctor, or other patients.

*I've talked to other patients. They were in the same situation. And they have always been positive with the surgery. They also chose the surgery. Mostly.* (Patient #10)

Most patients emphasized the importance of *trust in the medical care* in decision-making for or against surgery. This involved general trust in the clinical center in where the surgery was done, but also personal trust in the technical qualifications of the operating surgeon.

*Well, I wasn't scared in any way, that I would die on the operating table or something like that, I have incredible trust in the doctors here. I think that there are really good people here. [...] And that's why I never worried so much about it, regarding the whole operation, the anesthesia, the catheter, all that stuff. I knew, I was in good hands, and it was done well that way.* (Patient #3)

### Participation in an RCT

For the patients, *making a contribution to science* and the *hope that the novel therapy is superior to the established one*, to which they normally would not have access, were important reasons for *participating in an RCT*. However, the equality between the two treatment approaches was a frequently cited prerequisite for soing so.

*If the same goal or result, in inverted commas, comes out afterwards. Why not? (Patient #4)*

On the flip side, some patients stated that they would not want to participate in an RCT and emphasized the importance of actively choosing their treatment alongside their physician to feel safe. For them, chance should not play a role in decision-making.

*I would like to make up my mind, one way or the other. So, that there is a choice, right? But that depends on how affected you are and how large the tumor is. And it would be somehow helpful if a doctor said that you could do it like this or try it like that. So in someway together with the doctor. Because you cannot decide yourself. I mean, you usually have no previous experience, right?* (Patient #6)

## Discussion

This study highlights the open-mindedness of EC patients post neoadjuvant therapy towards both surgery on principle and close surveillance and surgery as needed. In this qualitative study, we interviewed patients after nCRT or nCT within the period pre- or post-surgery to achieve a broad spectrum of needs and preferences with respect to the potential options of active surveillance vs. immediate surgery. Patients demonstrated the ability to assess the pros and cons of each approach individually. However, there was significant heterogeneity in their judgments, emphasizing the need for an individualized approach in treatment discussions. This personalized approach, taking into account patient values and preferences, is crucial for informed decision-making.

Regarding surgery on principle, patients appreciated the security of tumor removal but expressed concerns about postoperative pain and potential reductions in quality of life (QoL), which align with findings from prior systematic reviews [25]. Interestingly, emotional functioning tended to improve post-surgery, potentially due to the sense of surviving a critical experience. The emphasis on QoL by interviewed patients resonates with studies indicating a preference for active surveillance over standard surgery among cancer patients [19]. The hypothesis that patients with depressed or anxious feelings are more likely to choose more radical treatments (in this case, surgery), as shown in other studies [26], was not examined in this study phase but will be investigated in the ESORES pilot study (DRKS00022050).

Research shows that while the fear of recurrence tended to decrease around the time of surgery, it often returned to baseline levels about a year after the operation (26). This temporal aspect should be considered in planning patient discussions. Since the survey period of our study extends to the weeks around the operation, and thus in the phase where the recurrence fear is presumably reduced, it can be assumed that the fear of recurrence would have been greater at other points in time.

Our results further imply that in discussions with the patients, physicians should bear in mind the patients’ exceptional situation—regarding emotions but also cognitive functioning. It was reported in our interviews that age-related impairments as well as side effects (e.g., “chemobrain”) affect the patients’ comprehension, memory, and reasoning. These cognitive impairments, as well as feelings of anxiety, shock, and uncertainty, should be considered in the information process. Using a more adapted language, allowing enough time to ask questions, and choosing suitable times of the day were suggested by the patients as possibilities to improve the quality of the discussions and increase the patient-centeredness of health care in general and clinical trials in particular.

The main reasons nominated for participating in RCTs were, on the one hand, that future patients or science will benefit, and on the other hand, that the novel therapy may be superior to the established one, meaning that the patients will benefit themselves. This concurrence of altruistic paired with egoistic intentions is sometimes refered to as *conditional altruism* in the literature and describes the initial willingness of patients to participate in a trial to help others, which will, however, only lead to participation if patients also recognize that they could benefit personally [27]. It is suggested that understanding and taking conditional altruism into account when planning an RCT can facilitate voluntary and informed patient inclusion. Knowing the reasons why cancer patients accept randomization and participate in RCTs should not be neglected, especially considering findings indicating that the acceptance for trials not providing active treatment in every arm might be significantly lower compared to trials providing active treatment in all arms of the study [28].

Even though many patients expressed their sometimes more, but often less concrete, fear about the operation, the short-term pain as a result of the operation was only a serious reason against the operation for very few patients. This, and the stated importance of long-term postoperative impairments in QoL, indicates a preference for long-term effects over short-term effects, which is also in line with a study that found out that short-term outcomes such as postoperative morbidity, surgeon’s reputation, and hospital type were less important to EC patients than overall survival rate and long-term QoL [18].

Strengths of our study include the heterogeneity of interviewed individuals, in terms of demographic characteristics and time to or since surgery. Contrary to this, a limitation of the current study is that all patients had a scheduled or accomplished esophagectomy. Thus, conducting a similar inquiry at a point in time when it is uncertain yet if the patients will choose esophagectomy or active surveillance would provide further valuable insights.

In summary, EC patients weigh *surgery on principle* and *active surveillance with surgery as needed* judiciously, necessitating tailored discussions that accommodate their cognitive and emotional challenges. The hope is that these findings encourage SDM and patient-centered healthcare, urging future studies to quantify these qualitative insights through randomized controlled trials, as it is the aim of the ESORES pilot study.

## Data Availability

No datasets were generated or analysed during the current study.
